# Syntaxin-1A Silencing by RNAi Disrupts Growth and Reproduction in the Asian Citrus Psyllid, *Diaphorina citri*

**DOI:** 10.3390/insects16090901

**Published:** 2025-08-28

**Authors:** Dingming Dong, Xingmin Wang, Baoli Qiu, Changqing Chang, Changfei Guo

**Affiliations:** 1 Engineering Research Center of Biocontrol, Ministry of Education and Guangdong Province, South China Agricultural University, Guangzhou 510642, China; gddongdingming@163.com (D.D.); wangxmcn@scau.edu.cn (X.W.);; 2 Key Laboratory of Green Prevention and Control of Agricultural Transboundary Pests of Yunnan Province, Agricultural Environment and Resource Research Institute, Yunnan Academy of Agricultural Sciences, Kunming 650205, China; 3 Integrative Microbiology Research Centre, College of Plant Protection, South China Agricultural University, Guangzhou 510642, China

**Keywords:** *Diaphorina citri*, Syntaxin-1A, RNAi, vitellogenin, pest control

## Abstract

*Diaphorina citri* is the primary global vector of “*Candidatus* Liberibacter asiaticus”, the bacterium responsible for Huanglongbing. Syntaxin-1A (*Syx1A*), a member of the Qa-SNARE family, is essential for vesicle fusion and signal transduction, though its function in hemipteran insects remains poorly understood. This study presents the first comprehensive analysis of *Syx1A* expression in *D. citri*. Transcripts were detected across all life stages, with peak expression in the salivary glands. RNAi silencing of *Syx1A* reduced mRNA levels by 39.0% in nymphs and 58.0% in adults, resulting in 58.3% nmortality in nymphs within 5 days and 73.3% in adults within seven days, along with significant weight loss. Treated females showed marked declines in fecundity, ovarian degeneration, and deficient yolk deposition. RT-qPCR confirmed significant downregulation of *Vg1*, *VgA*, and *VgR*. These findings establish *Syx1A* as a regulator of growth and reproduction in citrus psyllids via modulation of yolk synthesis. RNAi targeting of *Syx1A* represents a promising strategy for ecologically sound pest control and may contribute to efforts in halting the transmission of the Huanglongbing pathogen *C*Las.

## Simple Summary

The Asian citrus psyllid, *D. citri*, is a major vector of the citrus greening pathogen. This study identified Syntaxin-1A (*Syx1A*) as a gene expressed throughout all developmental stages of *D. citri*, with particularly high expression in the salivary glands. RNAi-mediated silencing of *Syx1A* resulted in 58% mortality in nymphs and 73% in adults, reduced body mass, inhibited oviposition, and led to pronounced ovarian atrophy. These effects are attributed to impaired regulation of yolk proteins critical for oogenesis. Thus, targeted suppression of *Syx1A* offers a species-specific and environmentally sustainable approach for controlling *D. citri* and limiting the spread of citrus greening disease.

## 1. Introduction

Huanglongbing (HLB), or citrus greening, is a phloem-restricted disease that threatens global citrus production. It is caused by the γ-proteobacterium “*Candidatus* Liberibacter asiaticus” (*C*Las), which infects all commercial citrus cultivars [1,2]. First identified in Guangdong Province, China, in the early 20th century, HLB was later confirmed in São Paulo, Brazil (2004), and Florida, USA (2005) [1,2]. Since then, it has rapidly spread across the USA, Mexico, Brazil, Belize, Puerto Rico, and Cuba, resulting in billions of dollars in economic losses [3,4]. Under natural conditions, HLB is primarily transmitted via grafting with infected scions and through the vector insect, the Asian citrus psyllid (*Diaphorina citri*). The rapid geographic expansion of *D. citri* throughout Asia and the Americas has significantly contributed to the widespread prevalence of HLB [2]. Currently, no effective method exists to fully eradicate HLB. The inability to detect *C*Las early and the rapid disease spread pose major challenges to control efforts [5]. Presently adopted management strategies include the use of disease-free seedlings, removal of infected trees, and suppression of the citrus psyllid vector [5]. Among these, *D. citri* control is considered one of the “three pillars” of integrated HLB management, alongside deploying pathogen-free nursery stock and eliminating infection reservoirs [6,7]. Chemical control remains the dominant approach for reducing *D. citri* populations, involving the extensive application of pyrethroid, organophosphate, neonicotinoid, and carbamate insecticides [8,9]. However, prolonged reliance on these pesticides has led to widespread resistance in psyllid populations [8,9,10,11,12,13]. In addition, frequent prophylactic applications pose ecological risks, including harm to non-target organisms and potential threats to human health [14]. These concerns underscore the urgent need for sustainable, alternative strategies to manage both *D. citri* and HLB.

RNA interference (RNAi) is a sequence-specific, post-transcriptional gene silencing mechanism that uses exogenous double-stranded RNA (dsRNA) to degrade target messenger RNAs (mRNAs) in pest organisms [15]. This approach enables precise gene function validation and targeted gene suppression of harmful insect species. Its efficacy has been demonstrated across more than 20 economically significant pest species from diverse orders, including Diptera, Coleoptera, and Hemiptera [16,17,18,19]. The utilization of RNAi technology extends beyond laboratory research, with its commercial applications increasingly penetrating the market. Presently, the primary agricultural application of RNAi technology involves pest control via the expression of double-stranded RNA (dsRNA) in genetically modified plants. This approach, termed Host-Induced Gene Silencing (HIGS), has been effectively implemented in a variety of crops [20,21]. Furthermore, non-transgenic RNAi products, such as Spray-Induced Gene Silencing (SIGS), are emerging as a prominent area of research due to their cost-effectiveness and operational simplicity [22]. However, several factors limit RNAi efficiency: dsRNA stability, insect uptake rates, intracellular RNAi machinery, and systemic spread [23]. For instance, in Lepidoptera insects, such as *Ostrinia nubilalis*, achieving efficient RNAi is challenging due to the instability of dsRNA in the gut and hemolymph, leading to its rapid degradation by enzymatic activity and a consequent significant reduction in RNAi efficiency [24]. Delivery method is a key determinant of success—while microinjection is suitable for laboratory studies, feeding and spraying are more practical for field applications. Yet in these methods, dsRNA is vulnerable to rapid degradation by nucleases [25,26]. To address this, researchers have developed nanocarriers such as cationic liposomes, chitosan, dendrimers (PDIs), carbon quantum dots (CQDs), and star-shaped polycations (SPc), which enhance both dsRNA stability and cellular uptake [27,28,29,30,31]. In citrus psyllid management, RNAi has shown considerable potential. Recent studies have successfully suppressed target gene expression and significantly reduced psyllid populations via feeding and spray-based dsRNA delivery; the genes targeted in these studies include Wing Disc (WD), cathepsin D, chitin synthase, apoptosis inhibitor *GS-K3*, hexokinase, *V-ATPase-E*, and *DcCP8*, among others [31,32,33,34,35,36,37]. Due to its high specificity, RNAi enables targeted silencing of essential genes using exogenously applied dsRNA, offering a novel, environmentally responsible, and sustainable alternative to chemical pesticides. Consequently, identifying optimal gene targets and refining sequence design are critical next steps for maximizing RNAi efficacy.

SNARE proteins act as molecular engines driving membrane fusion, thereby regulating cellular secretion and endocytosis, as well as participating in critical biological processes such as neurotransmission, autophagy, and tumorigenesis [38,39,40]. Functional differentiation among SNAREs is dictated by the presence of glutamine (Q-SNARE) or arginine (R-SNARE) residues within their core domains, enabling accurate targeting of membrane fusion sites and integration into broader signaling networks [41,42,43]. In *Drosophila melanogaster*, over 20 SNARE family members have been identified [44,45]. Among them, the Qa-SNARE Syntaxin 1A (Syx1A), discovered in 1995, is recognized as a multifunctional regulator [46]. Syx1A forms a ternary complex with VAMP2 and SNAP-25, facilitating synaptic vesicle fusion and neurotransmitter release [47,48]. Beyond its neuronal role, Syx1A is involved in embryonic membrane formation, cuticle hardening, and yolk metabolism [46]. Recent studies have further implicated Syx1A in regulating the midgut epithelial integrity of locust nymphs via the insulin/mTOR nutrient-sensing pathway [49].

Current research on the Syn1A gene in insects, aside from *Drosophila melanogaster* and *Locusta migratoria*, remains notably limited. In *Drosophila*, *Syn1A* has been characterized as a multifunctional regulatory factor involved in various processes, such as synaptic vesicle fusion, neurotransmitter release, cell growth, and protein transport [46,48]. Similarly, in locusts, *Syn1A* affects feeding behavior, midgut nutrient absorption, and ovarian yolk deposition by modulating the insulin/mTOR pathway, thereby directly influencing reproductive capacity [49,50]. Although these studies offer valuable insights into the function of *Syn1A* in certain insects, the role and potential functions of this gene in a broader spectrum of insect species warrant further investigation. Addressing this gap, the present study focuses on *Diaphorina citri* as a model organism. A comprehensive spatiotemporal expression profile of *Syx1A* was first mapped across diverse tissues and developmental stages. Subsequently, RNA interference (RNAi) was employed to effectively silence *Syx1A* expression. Knockdown of *Syx1A* significantly reduced the survival and reproductive capacity of *D. citri*, underscoring its essential role in growth, development, and fecundity. These findings position *Syx1A* as a promising RNAi target for the strategic suppression of citrus psyllid populations through the disruption of key physiological processes. This study thus provides both a robust molecular framework and a viable, environmentally sustainable approach for the targeted control of vector insects responsible for citrus greening disease.

## 2. Materials and Methods

### 2.1. Insects Rearing and Sample Preparation

A *D. citri* colony was continuously maintained at the Engineering Research Center of Biological Control, Ministry of Education, South China Agricultural University (Guangzhou, China). Insects were reared on orange jasmine (*Murraya exotica* L.) inside Plexiglas cages (60 × 60 × 60 cm) in a glasshouse maintained at 26 ± 1 °C, 55–65% RH, and a 14 L:10 D photoperiod. To synchronize cohorts, ~100 adults (5–10 days post-eclosion) were allowed to oviposit on fresh *M. exotica* shoots for 24 h before removal. Eggs were left to hatch, and nymphs developed on the host plant. Target instars and adults were gently collected using a fine camel-hair brush and aspirator, then snap-frozen at −80 °C. Each experiment was conducted with three biological replicates per treatment.

### 2.2. Identification of Syx1A and Bioinformatic Analysis

The full-length Syx1A cDNA was obtained from our in-house transcriptome (unpublished). Specific primers (Table S2), designed using Primer Premier 5, were used to amplify the open reading frame. PCR was performed in 25 µL reactions using LA Taq DNA polymerase (Takara, Shiga, Japan), following the cycling parameters described by Guo et al. [51]. PCR products were gel-purified, cloned into the pClone007 Blunt vector (Tsingke, Beijing, China), and sequenced. The deduced amino acid sequence was analyzed in MEGA 7.0, and conserved domains were annotated using SMART (http://smart.embl.de accessed on 15 July 2025) across multiple insect taxa and *Homo sapiens*. Multiple sequence alignments were conducted in DNAMAN, and a neighbor-joining phylogenetic tree was constructed in MEGA 7.0 with 1000 bootstrap replicates. GenBank accession numbers are listed in Table S1.

### 2.3. RNA Isolation and cDNA Synthesis

Total RNA was extracted using RNAiso Plus (Takara, Shiga, Japan) according to the manufacturer’s protocol. RNA purity and concentration were evaluated with a NanoDrop One Micro UV-Vis spectrophotometer (Thermo Fisher Scientific, Waltham, MA, USA). One microgram of RNA was reverse-transcribed into complementary DNA (cDNA) using the PrimeScript RT Reagent Kit with gDNA Eraser (Takara, Shiga, Japan), following the provided instructions. The resulting cDNA was stored at −80 °C for downstream applications, including conventional PCR, quantitative real-time PCR (qRT-PCR), and double-stranded RNA (dsRNA) synthesis.

### 2.4. RT-qPCR Analysis

Transcript quantification was performed using a CFX96 Real-Time System (Bio-Rad, Hercules, CA, USA), following the protocol described by Kong et al. [52]. Primer sequences are listed in Table S2, with *D. citri β-actin* serving as the endogenous reference gene [8]. Each cDNA sample was diluted 1:10, and 2.5 µL was used in a 15 µL qRT-PCR reaction comprising 7.5 µL SYBR Premix Ex Taq II (Tli RNaseH Plus, Takara, Shiga, Japan) and 0.3 µM of each primer. Reactions were run in triplicate using the following cycling conditions: initial denaturation at 95 °C for 30 s, followed by 40 cycles of 95 °C for 5 s and 55 °C for 30 s. Relative transcript levels were calculated using the 2^−ΔΔCt^ method. All assays included three biological replicates, each with three technical replicates.

### 2.5. Syx1A Expression Analysis of Different Developmental and Tissue

For each developmental stage, individuals were pooled per biological replicate as follows: 500 eggs, 300 first-instar, 150 second-instar, 75 third-instar, 40 fourth-instar, 20 fifth-instar nymphs, and 15 freshly emerged females plus 15 males. Three independent replicates were prepared for each stage. To analyze *Syx1A* expression, tissues were dissected from freshly emerged adults and fifth-instar nymphs under a stereomicroscope on ice in ice-cold 1× PBS. Adult tissues included midgut, salivary glands, hemolymph, testes, ovaries, bacteriome, antennae, legs, head, integument, fat body, and muscle. Nymphal tissues included the same, except with wing buds instead of reproductive organs. Each tissue pool consisted of 150 individuals, and three biological replicates were collected per tissue type. Samples were immediately placed in RNase-free 1.5 mL tubes containing 100 µL RNAiso Plus (Takara, Shiga, Japan), snap-frozen in liquid nitrogen, and stored at −80 °C until RNA extraction.

### 2.6. RNA Interference

To investigate the function of *Syx1A* in *Diaphorina citri*, RNA interference (RNAi) was employed. dsRNA templates were generated by PCR using gene-specific primers incorporating a 5′ T7 promoter sequence (5′-TAATACGACTCACTATAGGG-3′). GFP dsRNA served as a negative control. Each 50 µL PCR reaction contained 25 µL of 2× EasyTaq^®^ PCR SuperMix (+dye) (Sangon, Shanghai, China), 1 µL of cDNA, 2 µL of each 10 µM primer, and 20 µL of double-distilled water (ddH_2_O). PCR conditions were as follows: initial denaturation at 94 °C for 3 min; 35 cycles of 94 °C for 30 s, 55 °C for 30 s, and 72 °C for 45 s; followed by a final extension at 72 °C for 10 min. Amplified products were transcribed into dsRNA using the T7 RiboMAX™ Express RNAi System (Promega, Madison, WI, USA) per the manufacturer’s instructions. dsRNA concentration was determined with a NanoDrop 1000 spectrophotometer (Thermo Fisher Scientific, Waltham, MA, USA), and integrity was confirmed by agarose gel electrophoresis. Primer sequences are listed in Table S2.

### 2.7. Effect of Syx1A on the Survival Rate and Body Weight of Asian Citrus Psyllid

This study examined the effects of *Syx1A* knockdown on body mass and mortality in *Diaphorina citri* nymphs and adults. Freshly molted fifth-instar nymphs (<12 h post-molt) and newly emerged adults (1–2 days post-eclosion) were microinjected with 500 ng µL^−1^ of *dsSyx1A*. Each treatment group comprised over 120 individuals. A negative control group received dsGFP. Following injection, viable individuals were transferred to tender shoots of *M. exotica*. Gene silencing efficiency was assessed 48 h post-injection. Total RNA was extracted from whole insects and reverse-transcribed for RT-qPCR analysis. Four biological replicates were performed, each consisting of ten nymphs or ten adults. Mortality was recorded daily for nymphs (days 1–4) and every other day for adults (days 1, 3, 5, 7). Body mass was measured on day 3 for nymphs and day 5 for adults.

### 2.8. Effect of on Syx1A Female Fecundity

To evaluate the effect of *Syx1A* knockdown on female fecundity, *dsSyx1A*-injected females were paired 1:1 with age-matched males on fresh *M. exotica* shoots to ensure normal mating and oviposition. dsRNA injections were performed as previously reported [53,54]. And drawing on these studies, along with our previous experimental data, we concluded that a dsRNA concentration of 500 ng/µL achieves optimal RNAi efficiency in *D. citri* for most target genes. Beginning on day 3 post-injection—when oviposition commenced—shoots were replaced every 48 h. Eggs laid on removed shoots were counted to quantify per-female fecundity through day 11, ensuring sustained RNAi activity throughout the reproductive window. For ovarian phenotyping, females from both treatment groups (*dsSyx1A* and *dsGFP*) were dissected two days after mating. Ovaries were excised in sterile 1× PBS under a stereomicroscope and examined for morphological differences. Three independent biological replicates were conducted.

### 2.9. Effect of Syx1A Silencing on the Expression of Vitellogenin and Its Receptor Genes

The experiments revealed that *Syx1A* suppression significantly impaired oviposition and ovarian development, implicating this gene in oogenesis. Since vitellogenin (*Vg*) and its receptor (VgR) are essential for insect reproduction, we assessed transcript levels of *D. citri Vg1*, *VgA*, and *VgR* to determine the impact of *Syx1A* knockdown. On day 3 post-injection, total RNA was extracted from adult females and analyzed via RT-qPCR as previously described. Primer sequences are listed in Table S2.

### 2.10. Statistical Analysis

Data were analyzed using SPSS software version 18.0 (SPSS Inc., Chicago, IL, USA). Expression patterns of *Syx1A* were evaluated using one-way ANOVA, with statistical significance set at *p* < 0.05. Tukey’s multiple range test was employed for pairwise comparisons. Independent-samples *t*-tests were used to analyze gene expression, body weight, and egg production, also with a significance threshold of *p* < 0.05. All experiments included a minimum of three biological replicates.

## 3. Results

### 3.1. Sequence Characterization and Phylogenetic Insights into Syx1A

**Figure 1:**
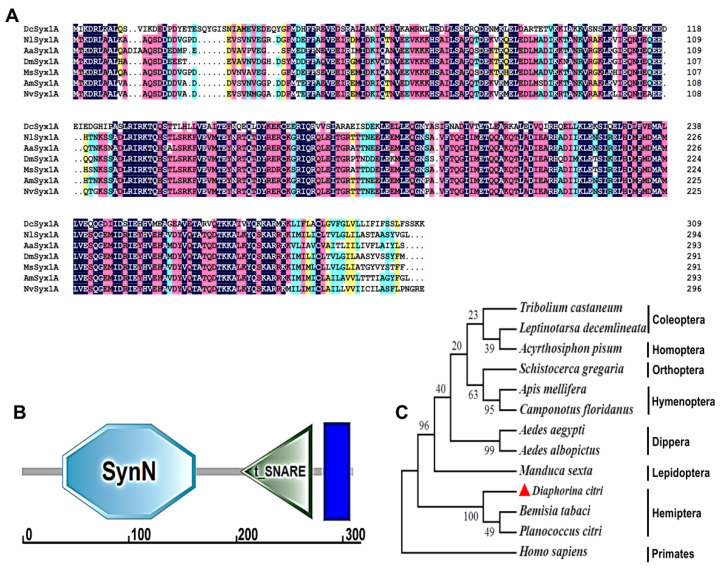
Bioinformatics analysis of *Syx1A* in *Diaphorina citri*. (**A**) Multiple sequence alignment of Syx1A proteins from different species, including *Diaphorina citri* (Dc), *Nilaparvata lugens* (Nl), *Aedes albopictu* (Ab), *Drosophila melanogaster* (Dm), *Manduca sexta* (Ms), *Apis mellifera* (Am), and *Nasonia vitripennis* (Nv). Color-coded sequence identity is displayed as follows: perfect matches (100%) in blue, strong similarity (≥75%) in red, moderate similarity (≥50%) in green, and weak similarity (≥30%) in yellow. (**B**) Structural domain analysis of the *Syx1A* protein. (**C**) Phylogenetic analysis of *Syx1A* proteins across different insect species and *Homo sapiens* using the neighbor-joining method in MEGA 7.0. *Syx1A* protein sequences from the various insect species examined, together with their GenBank accession numbers, are provided in Table S1. *Syx1A* is marked with red triangles.

From the *Diaphorina citri* transcriptome, we successfully cloned the full-length open reading frame of *Syx1A*, comprising 930 nucleotides. This sequence encodes a 309-amino-acid protein with a predicted molecular mass of 35.72 kDa and an isoelectric point of 5.104. The deduced protein features a canonical SynN domain (amino acids 38–162), a SNARE domain (204–271), and a transmembrane region (283–305), consistent with the typical domain architecture of insect Syx1A proteins (Figure 1B). Multiple sequence alignment revealed that the SNARE domain is highly conserved among insects, with 98–100% identity, while the full-length *D. citri* Syx1A shares 78.85% identity with orthologues from other species (Figure 1A). Phylogenetic analysis showed that *Syx1A* proteins from diverse insect orders form a monophyletic clade, with the *D. citri* sequence clustering closely with hemipteran homologues from *Bemisia tabaci* and *Planococcus citri* (Figure 1C).

### 3.2. Expression Profiles of Syx1A Across Developmental Stages and Tissues

**Figure 2:**
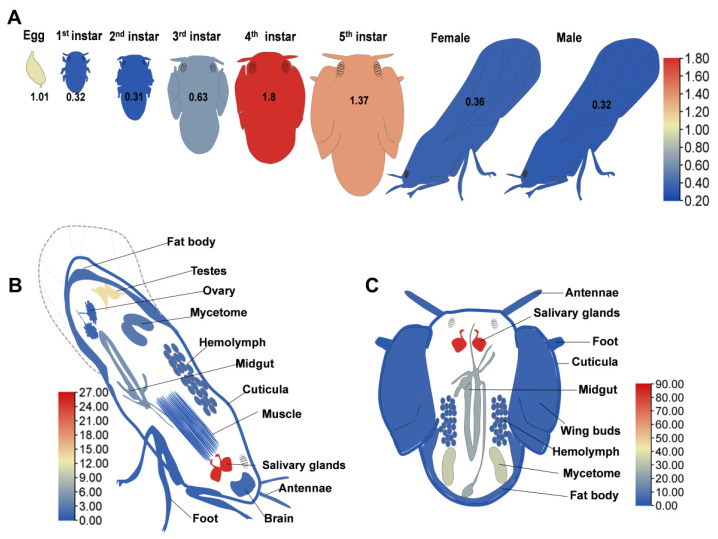
Expression profiles of *Syx1A* in different life stages of Diaphorina citri and in various tissues of adults and nymphs. (**A**) Temporal expression of *Syx1A* across developmental stages. (**B**) Tissue-specific expression of *Syx1A* in adult females and males. (**C**) Tissue-specific expression of *Syx1A* in fifth-instar nymphs. Relative expression levels were calculated using 2^−ΔΔCt^ method. The expression profile of Syx1A is displayed as a heat map (main Figure 2) and a bar chart (Figure S1); color intensity corresponds to expression level, as indicated by the scale bar. All quantitative analyses were performed in SPSS and are presented as mean ± SE. Based on three biological replicates (each with three technical replicates), one-way ANOVA followed by Tukey’s post hoc test was applied; different letters denote statistically significant differences among developmental stages (*p* < 0.05).

To characterize Syx1A expression, RT-qPCR was performed across all developmental stages of *D. citri*, from eggs and 1st–5th instar nymphs to newly emerged adult males and females. *Syx1A* transcripts were detected at every stage, with the highest levels in 4th and 5th instar nymphs, followed by eggs and 3rd instar nymphs. No significant differences were observed between transcript levels in the 1st and 2nd instars and adults (Figure 2A and Figure S1A). Tissue-specific expression analyses were subsequently conducted on 5th instar nymphs and adults within five days post-eclosion. *Syx1A* expression displayed strong tissue specificity in both life stages. In nymphs, transcript abundance was highest in the salivary glands, followed by the mycetome and midgut, with low expression in the cuticle, antennae, wing buds, hemolymph, fat body, and feet (Figure 2C and Figure S1C). In adults, salivary glands again showed the highest expression, followed by the testes and midgut, while the cuticle, antennae, mycetome, ovaries, head, hemolymph, fat body, and feet exhibited moderate levels. Muscle tissues showed the lowest expression (Figure 2B and Figure S1C).

### 3.3. Evaluation of dsRNA-Mediated Silencing Efficiency of Exogenous Syx1A in Nymphs and Adults of Diaphorina citri

**Figure 3:**
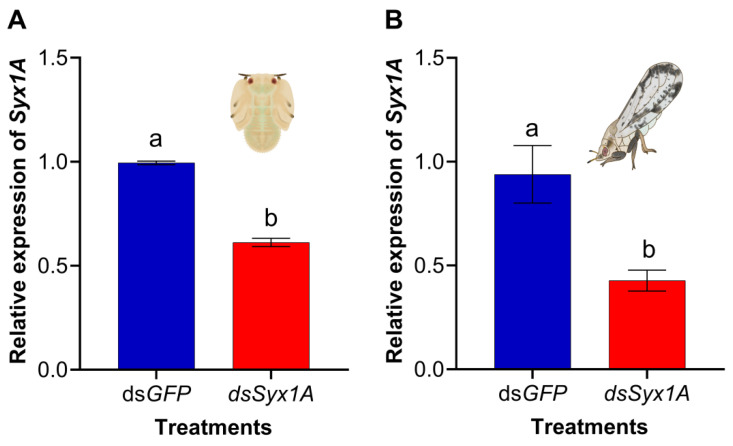
RNAi efficiency of *dsSyx1A* in Asian citrus psyllid nymphs (**A**) and adults (**B**). Nymphs and adults were separately injected with *dsSyx1A* or the control *dsGFP*, and samples were collected 48 h later. Relative expression levels were calculated using 2^−ΔΔCt^ method. Data are expressed as mean ± SE from three biological replicates; inter-group differences were analyzed with an independent-samples *t*-test. Different letters denote significant differences between the treatment and control groups (*p* < 0.05).

To assess gene silencing efficiency, newly molted 5th instar nymphs (<12 h post-molt) and synchronously emerged adults were microinjected with 500 ng µL^−1^ of *dsSyx1A*; *dsGFP* served as a negative control. RT-qPCR at 48 h post-injection confirmed a significant knockdown of Syx1A expression: 39.0 ± 2.8% reduction in nymphs (Figure 3A) and 58.0 ± 3.1% in adults (Figure 3B). These results demonstrate that dsRNA treatment effectively and consistently suppresses Syx1A expression in *D. citri*.

### 3.4. Effects of Syx1A Silencing on Body Weight and Survival of Diaphorina citri Nymphs and Adults

**Figure 4:**
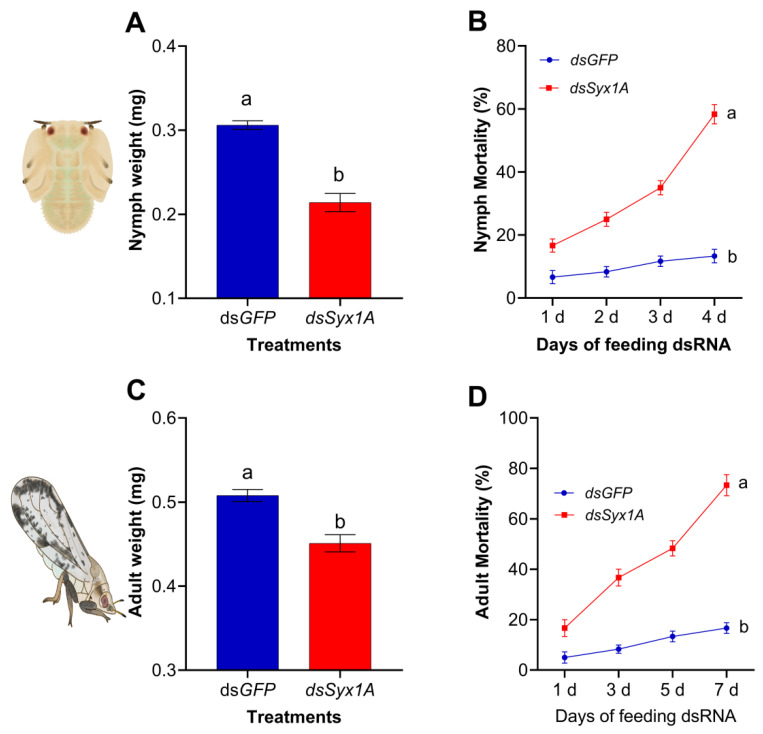
Impact of *Syx1A* RNAi on the body weight and mortality of *Diaphorina citri.* (**A**,**B**) show weight and mortality in 5th-instar nymphs following *Syx1A* knockdown, whereas panels (**C**,**D**) present the corresponding data for adults. Values are mean ± SE; different letters denote significant differences between treatment and control groups (independent-samples *t*-test, *p* < 0.05).

To investigate the functional role of *Syx1A* in *D. citri* development, RNAi experiments were conducted on 5th instar nymphs and newly emerged adults. Survival monitoring revealed significantly increased mortality in dsSyx1A-treated groups: nymph mortality reached 58.3% by day 5 compared to 13.3% in the *dsGFP* group (Figure 4B), while adult mortality rose to 73.3% by day 7, far exceeding the 16.6% observed in controls (Figure 4D). In parallel, body-weight measurements showed significantly reduced weights in both nymphs and adults following *dsSyx1A* treatment compared to controls (Figure 4A,D). Collectively, these findings underscore the essential role of *Syx1A* in the growth, development, and survival of *D. citri*.

### 3.5. Impact of Syx1A Silencing on Oviposition and Ovarian Development in Adult Diaphorina citri

**Figure 5:**
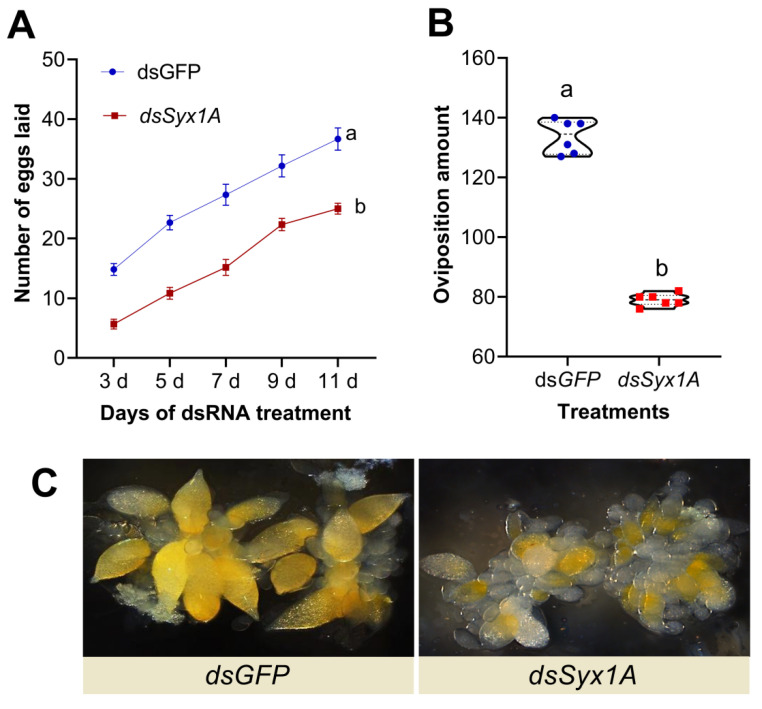
Impact of *Syx1A* silencing on reproduction and ovarian development in *Diaphorina citri*. (**A**) Daily fecundity per female following *Syx1A* silencing. (**B**) Cumulative 8-day fecundity per female after *Syx1A* silencing. (**C**) Morphological effects of *Syx1A* knockdown on ovary development in adult females. Values are mean ± SE; different letters denote significant differences between treatment and control groups (independent-samples *t*-test, *p* < 0.05).

Silencing of Syx1A via RNA interference (RNAi) significantly impaired female reproductive performance. Daily fecundity analysis revealed that, beginning on day 3, egg production in the *dsSyx1A* group consistently lagged behind that of the *dsGFP* controls, with statistically significant reductions observed at five consecutive time points (days 3, 5, 7, 9, and 11; Figure 5A). As a result, cumulative egg production over the 8-day monitoring period was markedly reduced (Figure 5B), indicating that Syx1A deficiency disrupts oocyte maturation and oviposition. Ovarian morphology supported this interpretation: by day 3, ovaries from dsGFP females contained ovarioles filled with mature, yellow-pigmented oocytes, consistent with normal vitellogenesis. In contrast, ovaries from *dsSyx1A* females were visibly smaller, exhibited diminished vitellin deposition, and harbored oocytes arrested at early to mid-developmental stages (Figure 5C). Collectively, these results establish that *Syx1A* is essential for vitellogenesis and ovarian development and is therefore critical for reproductive success in *D. citri*.

### 3.6. Influence of Syx1A Silencing on the Expression of Vg1, VgA, and VgR in Adult Diaphorina citri

**Figure 6:**
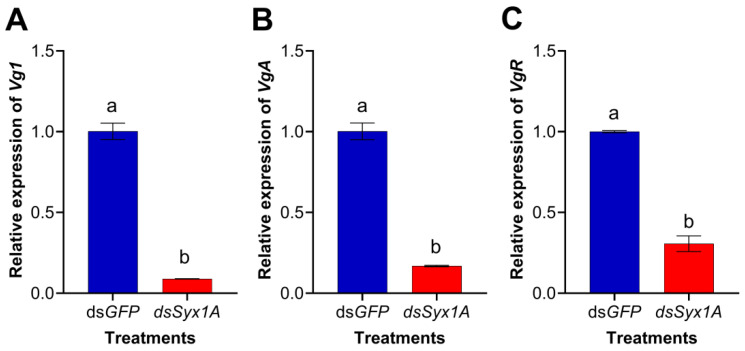
Effects of *Syx1A* RNAi on the expression of *Vg1*, *VgA*, and *VgR* in the adult *Diaphorina citri*. (**A**) *Vg1*, (**B**) *VgA*, and (**C**) *VgR* transcriptional response to *Syx1A* knockdown. The 2^−∆∆Ct^ method was adopted to calculate the relative expression level. Data are presented as mean ± SE. Different letters denote significant differences between treatment and control groups (independent-samples *t*-test, *p* < 0.05).

To investigate the molecular basis underlying Syx1A-mediated reproductive regulation, we quantified key vitellogenic transcripts three days after *dsRNA* treatment. Compared to *dsGFP* controls, transcript levels of *Vg1*, *VgA*, and *VgR* were significantly reduced in *dsSyx1A* females (Figure 6A–C). This coordinated downregulation suggests that *Syx1A* acts upstream of both vitellogenin synthesis (*Vg1*/*VgA*) and receptor-mediated uptake (*VgR*). Thus, Syx1A silencing decreases vitellogenin availability and hinders its ovarian sequestration, leading to arrested oocyte maturation and reduced egg production. These findings demonstrate that *Syx1A* regulates female reproductive output in *D. citri* by modulating the expression of vitellogenic genes.

## 4. Discussion

In *D. melanogaster*, *Syx1A* has been characterized as a multifunctional regulator involved in synaptic vesicle fusion, neurotransmitter release, cell growth, and protein trafficking [46,48]. Similarly, in *L. migratoria*, *Syx1A* influences feeding behavior, midgut nutrient absorption, and ovarian yolk deposition through modulation of the insulin/mTOR pathway, directly impacting reproductive capacity [49,50]. Despite these insights, the expression dynamics, functional networks, and hormonal regulation of *Syx1A* across other insect species remain poorly understood, highlighting the need for broader comparative analyses.

This study is the first to isolate and clone the full-length cDNA of Syx1A from *D. citri*. Bioinformatic analysis revealed the presence of a SynN domain at the N-terminus and a canonical SNARE domain located proximally to the transmembrane region. Cross-species alignment of the SNARE motif showed high conservation within the insect class, suggesting strong evolutionary pressure to maintain its functional integrity [39,40,41,48]. All SNARE proteins harbor a conserved SNARE motif that adopts a parallel four-helix bundle structure; in synaptic SNARE complexes, SNAP-25 contributes two motifs, while Syntaxin and Synaptobrevin each contribute one, collectively forming a functional unit [55]. The conserved sequence and domain architecture of *Syx1A* across insects strongly support its role as a core component in membrane fusion machinery.

In *L. migratoria*, *Syx1A* is consistently expressed at high levels throughout all developmental stages [49,50], a pattern corroborated in this study for *D. citri*, suggesting that Syx1A is critical across the hemipteran life cycle. In Drosophila, *Syx1A* is essential for salivary gland vesicle dynamics during the pupal stage, ensuring proper glandular function [56]. Notably, although *Syx1A* mRNA levels are low in larval salivary glands, the protein localizes abundantly to the apical membrane of late-stage larvae [57], indicating high protein stability and recycling post-exocytosis via endocytosis and late endosome fusion. In *D. citri*, *Syx1A* is ubiquitously expressed across all examined tissues but is particularly elevated in the salivary glands of both nymphs and adults. This expression pattern contrasts with that of *Drosophila* and may reflect the continuous requirement for effector protein secretion by the salivary glands to suppress plant defense responses and enable sustained phloem feeding. The high transcript abundance of *Syx1A* likely meets the increased demand for vesicle trafficking and membrane fusion involved in this process [58,59,60]. Thus, the pronounced expression of *Syx1A* in *D. citri* salivary glands may represent an adaptive evolutionary strategy aligned with its herbivorous, phloem-feeding lifestyle.

RNA interference (RNAi) has been extensively validated as a functional genomics tool in hemipteran insects, with microinjection recognized as the most reliable method for delivering double-stranded RNA (*dsRNA*) due to its precise dosage control and high systemic diffusion efficiency [36,37,49,61]. The *Diaphorina citri* genome contains all core components of the RNAi machinery, and previous studies have demonstrated successful gene silencing using RNAi-based approaches [32]. In this study, dsRNA targeting *Syx1A* (*dsSyx1A*) was microinjected into fifth-instar nymphs and newly emerged adults. Quantitative RT-PCR analysis confirmed that *Syx1A* expression was reduced by 39.0% and 58.0% in nymphs and adults, respectively. These results confirm that *dsRNA* delivery via microinjection effectively induces stable and efficient gene silencing in *D. citri*, thereby establishing a technical foundation for investigating the functional role of *Syx1A* in the insect’s development and reproduction.

Intestinal epithelial cells facilitate digestion by secreting hydrolytic enzymes such as trypsin, carboxypeptidase, and aminopeptidase [62]. Disruption of midgut protease gene function impairs digestive efficiency and nutrient absorption [63]. In *Locusta migratoria*, microinjection of dsRNA targeting *LmSyx1A* led to complete mortality in fifth-instar nymphs, accompanied by significant reductions in food intake and body weight [49]. Histological analyses revealed damage to the brush border and disrupted columnar epithelial structure, highlighting the essential role of *Syx1A* in maintaining midgut integrity [49]. Building on these findings, the present study demonstrates that *Syx1A* silencing in *D. citri* significantly reduces body weight in both nymphs and adults, with mortality rates of 58.3% and 73.3%, respectively. These outcomes suggest that *Syx1A* is critical for nutrient assimilation and growth, providing direct evidence for its utility as a target in RNAi-based pest control strategies.

The involvement of syntaxin proteins in reproduction has been demonstrated in multiple arthropods. For instance, SNAP-25 is essential for reproduction in the tick *Amblyomma maculatum* [64], while *Syx1A* regulates ovarian development and reproductive output in both *D. melanogaster* and *L. migratoria* [46,50]. Given the high evolutionary conservation of Syx1A, its role in reproduction is likely conserved across insect species. Consistent with this, *Syx1A* silencing in *D. citri* significantly reduced egg production. Ovarian dissections revealed impaired yolk deposition in the *dsSyx1A*-injected group, whereas ovaries in the control group developed normally. These findings indicate that *Syx1A* is essential for female sexual maturation, likely by facilitating yolk accumulation within developing oocytes.

Vitellogenin (*Vg*), the precursor of yolk proteins, is synthesized primarily in the fat body and serves as a key marker of reproductive capacity in insects [65]. In locusts, *Syx1A* is highly expressed in the fat body, and its knockdown significantly reduces Vg transcript levels, implicating *Syx1A* in nutrient storage and energy metabolism related to reproduction [49,50]. A comparable expression pattern was observed in *D. citri*, where silencing *Syx1A* in the fat body led to the downregulation of *Vg1*, *VgA*, and their receptor *VgR*. Previous studies have shown that silencing Vg1/VgA/VgR compromises reproductive output in *D. citri* [66,67]. Based on these observations, we propose that *Syx1A* regulates reproductive function by modulating *Vg* synthesis in the fat body and Vg uptake in the ovaries—an evolutionarily conserved mechanism among hemipteran insects.

## 5. Conclusions

In conclusion, this study demonstrates that *Syx1A* plays a pivotal role in the growth, development, and reproduction of *D. citri*. RNAi-mediated silencing of *Syx1A* impairs weight gain, increases mortality, and disrupts reproductive processes by downregulating *Vg1*/*VgA*/*VgR* expression, thereby inhibiting yolk synthesis and uptake. These findings establish *Syx1A* as a promising molecular target for RNAi-based pest management strategies aimed at suppressing *D. citri* populations and limiting the spread of Huanglongbing. This work lays the groundwork for the development of sustainable, gene-targeted control methods against this critical citrus pest.

## Data Availability

Data are contained within the article or in the Supplementary Materials. All scripts used for data processing, statistical modeling, and figure generation are available from the corresponding author upon reasonable request. Further inquiries can be directed to the corresponding author.

## Supplementary Materials

The following supporting information can be downloaded at https://www.mdpi.com/article/10.3390/insects16090901/s1. Table S1: Species and GenBank accession numbers of amino acid sequences used for conducting phylogenetic tree; Table S2: Primer sequences used for RNAi and RT-qRCR in this study; Figure S1: Expression of *Syx1A* at different developmental stages and tissues of *Diaphorina citri* using RT-qPCR; Figure S2: The heatmap visualizes the developmental stage and tissue specific expression profile of the *Syx1A* gene in *Diaphorina citri*; Figure S3. Nucleotide and deduced amino acid sequences of *Syx1A* genes of *Diaphorina citri*.
