# Supplementary material for: Syntaxin-1A Silencing by RNAi Disrupts Growth and Reproduction in the Asian Citrus Psyllid, Diaphorina citri
Source: Insects. 2025 Aug 28;16(9):901. doi: 10.3390/insects16090901 (PMC12470858; doi:10.3390/insects16090901)
Supplement: Supplementary file 1 [file insects-16-00901-s001.zip › insects-3826581-supplementary.pdf]

Supplementary table

Table S1. Species and GenBank accession numbers of amino acid sequences used for conducting phylogenetic tree.

Order	Species	GenBank accession number
Diptera	*Aedes aegypti*	XP_021695761.1
	Aedes albopictu	XP_029716663.1
	Drosophila melanogaster	NP_524475.1
Lepidoptera	*Manduca sexta*	XP_037295517.1
Homoptera	*Acyrthosiphon pisum*	NP_001155922.1
Hymenoptera	*Apis mellifera*	NP_001265255.1
	Nasonia vitripennis	XP_016839192.1
	Camponotus floridanus	XP_019882021.1
Coleoptera	*Tribolium castaneum*	KYB25401.1
	Leptinotarsa decemlineata	XP_023011687.1
Hemiptera	*Bemisia tabaci*	XP_018896672.1
	Nilaparvata lugens	XP_022196535.1
	Planococcus citri	XP_065215190
Orthoptera	*Schistocerca gregaria*	XP_049858696.1
Primates	*Homo sapiens*	AAA53519.1

Table S2. Primer sequences used for RNAi and RT-qRCR in this study

Primer name	Sequence (5'-3')	Application
ds*Syx1A*-F	taatacgactcactatagggAGCGATCTCCTCTCTTCCCC	RNAi
ds*Syx1A*-R	taatacgactcactatagggTGTACCCGTGCTGTCTCAAC	
ds*GFP*-F	taatacgactcactatagggAAGTTCAGCGTGTCCGGCGAGG	
ds*GFP*-R	taatacgactcactatagggTTCACCTTGATGCCGTTCTTC	
q*Syx1A*-F	CACGTCATGGAAGCTGGAG	RT- qPCR
q*Syx1A*-R	GGCAGGCGAGAAATATCAGC	
CDS-*Syx1A-F*	ATGATCAAGGATAGACTGAAAGCAC	ORF
CDS-*Syx1A-R*	TTATTTCTTGGACGAGAATAAACTGGA	
β-actin-F	CCCTGGACTTTGAACAGGAA	
β-actin-R	CTCGTGGATACCGCAAGATT	
GAPDH-F	TGAGATCAAGGCCAAGGTAAAG	
GAPDH-R	GTCAAAGATGGAGGAGTGAGTG	
qVg1-F	GCTCAGATCTACGCTCTGCC	
qVg1-R	TTTCTCCAGTGAAAGATCCACA	
qVgA-F	CCAACTTCCAACGTCTTTCA	
qVgA-R	GTAATTCGTGGCTTTCTCCG	
qVgR-F	CCTGATTGTACTGGGGGAGA	
qVgR-R	AAAACTCGGAACATGGCAAC	

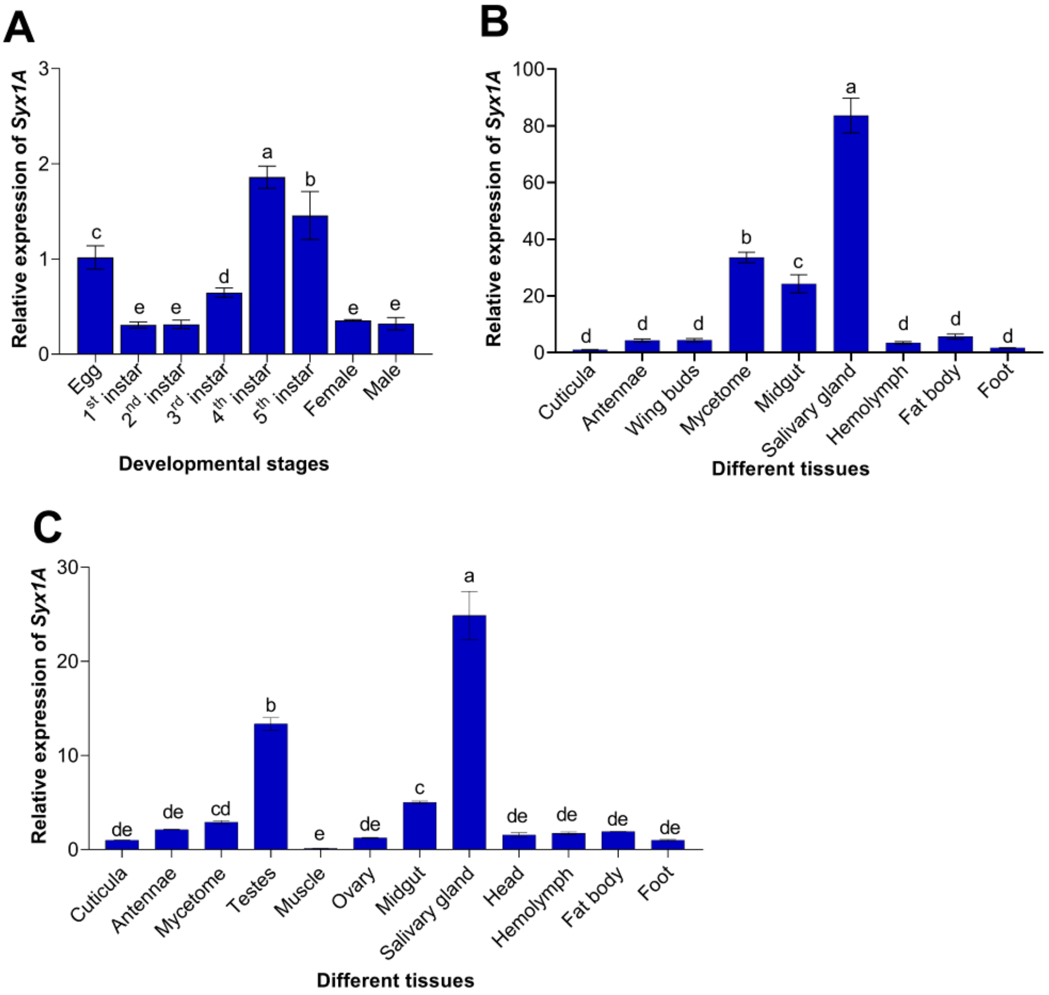

Figure S1. Expression of *Syx1A* at different developmental stages and tissues of *Diaphorina citri* using RT-qPCR. (A) Expression of *Syx1A* in different developmental stages. (B) Expression of *Syx1A* in different tissues of adults. (C) Expression of *Syx1A* in different tissues of 5thinstar nymphs. Relative expression levels were calculated using $2^{-\Delta\Delta Ct}$ method. All data analysis were performed using SPSS software. Data are means ± standard error (SE). Different letters indicate significant differences among developmentalstages, based on 3 biological replications and 3 technical repeats($P < 0.05$; analysis of variance, Tukey's multiple comparisonstest).

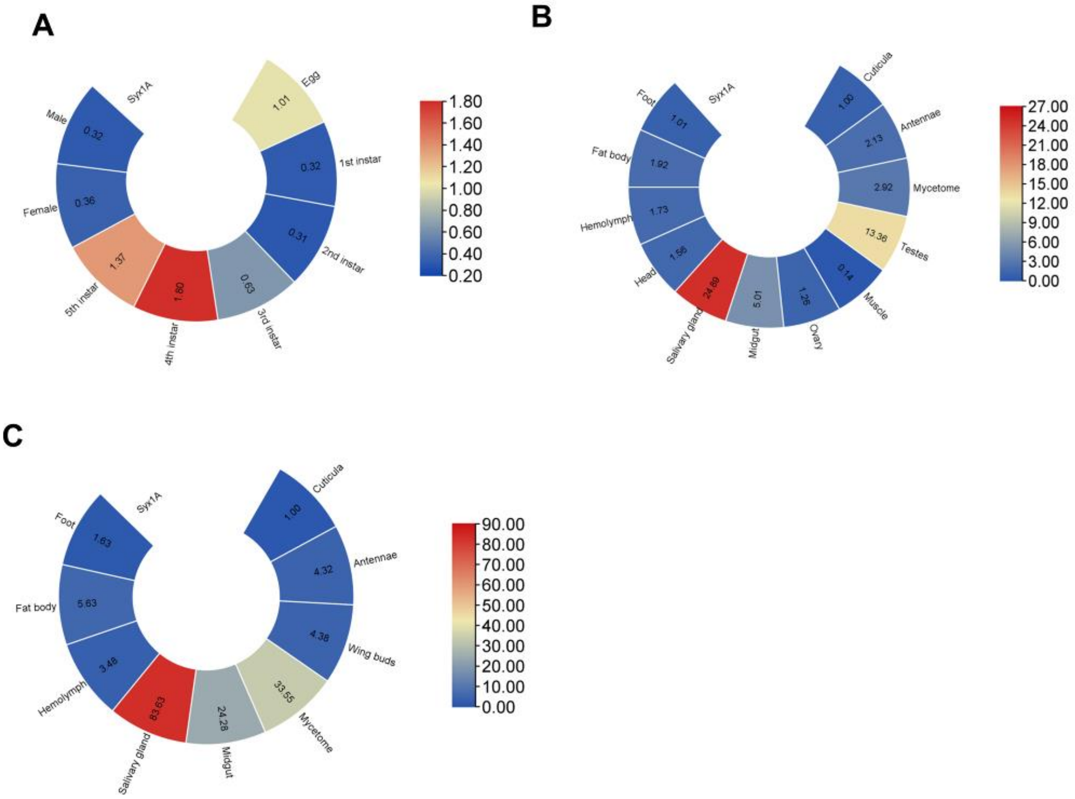

Figure S2. The heatmap visualizes the developmental stage and tissue specific expression profile of the Syx1A gene in *Diaphorina citri*. The color scale represents Syx1A expression. The range of the color gradient is indicated in each respective panel.

```
1   ATGATCAAGGATAGACTGAAAGCACTCCAGTCCGTTATCAAAGATGAAGACCCAGACTACGAGACGGAGAGTCAGTATGGAATCTCCAATATCGCCATGGAGGTTGAAGACGAACAGTAT
     M  I  K  D  R  L  K  A  L  Q  S  V  I  K  D  E  D  P  D  Y  E  T  E  S  Q  Y  G  I  S  N  I  A  M  E  V  E  D  E  Q  Y
121 GGATTCATGGACCACTTCTTTCGAGAGGTTGAAGACAGTCGAGCGCTCATCGCCAACATTCAAGAACACGTGAAAGCGATGCGAAATCTTCACAGCGATCTCCTCTCTTCCCCGCGACAA
     G  F  M  D  H  F  F  R  E  V  E  D  S  R  A  L  I  A  N  I  Q  E  H  V  K  A  M  R  N  L  H  S  D  L  L  S  S  P  R  Q
241 GACGAAAATATGAAGTTGGAGCTCGATGCTCGTACTGAGACAGTGAAGAAAATCGCGAAGAAAGTGAGCAATAGTCTGAAAAAGCTGGAGCGGAGTATAAAGAAGGAAGAGGATGAAATA
     D  E  N  M  K  L  E  L  D  A  R  T  E  T  V  K  K  I  A  K  K  V  S  N  S  L  K  K  L  E  R  S  I  K  K  E  E  D  E  I
361 GAGGATGGGCACATTCCCGCCAGTCTGAGGATTCGGAAGACACAACAGTCCACCACCTTACACCTCCTGGTAGAAGCTATCACGGAATTCAACCAGGAGCAGCTAGACTATAAGGAAAAA
     E  D  G  H  I  P  A  S  L  R  I  R  K  T  Q  Q  S  T  T  L  H  L  L  V  E  A  I  T  E  F  N  Q  E  Q  L  D  Y  K  E  K
481 TGTGAAGAAAGAATACAGCGTGTGGTATCCATAGCCAGAGCAGAGATCTCAGATGAGAAGCTAGAAGAACTGCTGGAGCAAGGAAACTATGCGTCTATATTCAATGCTGACATCGTGACT
     C  E  E  R  I  Q  R  V  V  S  I  A  R  A  E  I  S  D  E  K  L  E  E  L  L  E  Q  G  N  Y  A  S  I  F  N  A  D  I  V  T
601 GAAACTCTGGAAGCAAGGAAGGCGCTGGAAGATGTACAGATTAGACATCAAGAGTTACTCAAACTGGAGAAATCCATACAGGAACTACGCGACCTGTTTGTGGAAATGGCGCTACTTGTA
     E  T  L  E  A  R  K  A  L  E  D  V  Q  I  R  H  Q  E  L  L  K  L  E  K  S  I  Q  E  L  R  D  L  F  V  E  M  A  L  L  V
721 GAACAGCAGGGTGATATTATTGACAGTATTGAACATCACGTCATGGAAGCTGGAGAGGCGGTTGAGACAGCACGTGTACAAACCAAGAAGGCCATCGTGTATCAGAAGAAAGCTAGAATG
     E  Q  Q  G  D  I  I  D  S  I  E  H  H  V  M  E  A  G  E  A  V  E  T  A  R  V  Q  T  K  K  A  I  V  Y  Q  K  K  A  R  M
841 AAAAAGCTGATATTTCTCGCCTGCCTTGGGGTATTCGGACTGGTTCTACTCATCTTCATTTTCTCCAGTTTATTCTCGTCCAAGAAATAA 930
     K  K  L  I  F  L  A  C  L  G  V  F  G  L  V  L  L  I  F  I  F  S  S  L  F  S  S  K  K  *
```

Figure S3. Nucleotide and deduced amino acid sequences of Syx1A genes of *Diaphorina citri*. The start (ATG) and stop (TAA) codons are marked with rectangle boxes.

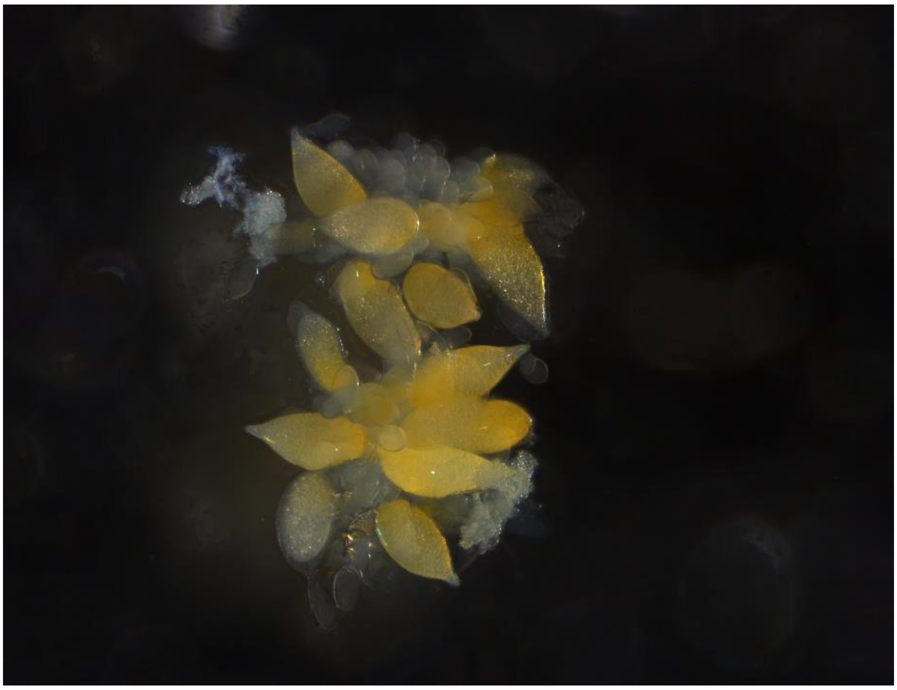

Morphological effects of ***dsGFP*** on ovary development in adult females.

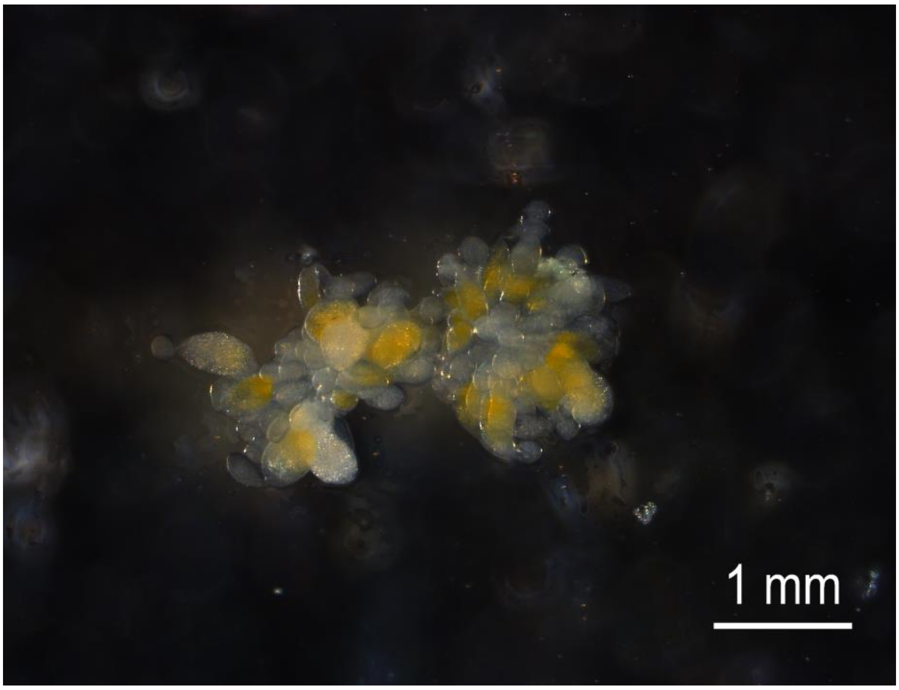

Morphological effects of ***Syx1A*** knockdown on ovary development in adult females.
